# Premacular Subhyaloid Hemorrhage in a Young Adult After Cannabis Consumption: A Case Report

**DOI:** 10.7759/cureus.105301

**Published:** 2026-03-16

**Authors:** Ines Douillet, Ayman Mabchour

**Affiliations:** 1 Ophthalmology, Erasmus Hospital, Brussels, BEL

**Keywords:** cannabis consumption, nd:yag laser hyaloidotomy, premacular subhyaloid hemorrhage, retinal hemorrhage, retinal vascular events, young adult

## Abstract

Premacular subhyaloid hemorrhage is an uncommon cause of acute visual impairment and is most frequently associated with Valsalva, trauma, hypertension, retinal vascular disease, or hematologic disorders. However, idiopathic cases have been described, suggesting the possibility of additional contributing factors.

We describe the case of a 25-year-old man who presented with sudden unilateral visual loss two hours after cannabis consumption. Clinical examination and multimodal imaging confirmed a large premacular subhyaloid hemorrhage in the absence of trauma, Valsalva maneuver, systemic disease, or underlying retinal vascular abnormalities.

Given the macular location and large size of the hemorrhage, Nd:YAG laser hyaloidotomy was performed to facilitate drainage of blood into the vitreous cavity and accelerate visual recovery. Laser energy was progressively increased from low initial settings until successful perforation of the posterior hyaloid was achieved at 10 mJ, resulting in immediate drainage of the hemorrhage. The procedure was well tolerated without complications, and best-corrected visual acuity returned to 20/20 (Snellen) one week after treatment.

This case highlights the value of Nd:YAG laser hyaloidotomy for selected large premacular hemorrhages and emphasizes technical considerations when performing the procedure in young patients, in whom greater posterior hyaloid thickness and adherence may require higher laser energy. It also describes a premacular hemorrhage occurring in temporal proximity to cannabis use. Although causality cannot be established, the observation raises the hypothesis that cannabis-related vascular effects could potentially contribute to retinal hemorrhagic events.

## Introduction

Subhyaloid hemorrhage is an accumulation of blood in the preretinal space, between the posterior hyaloid face and the internal limiting membrane (ILM). When the hemorrhage involves the macular region, it can cause sudden painless visual loss due to obstruction of the fovea [1]. Clinically, these hemorrhages often appear as well-demarcated “boat-shaped” or dome-shaped preretinal collections [2].

Premacular subhyaloid hemorrhage is relatively uncommon and is frequently associated with Valsalva maneuver, trauma, proliferative diabetic retinopathy, retinal macroaneurysm, age-related macular degeneration, polypoidal choroidal vasculopathy, and hematologic disorders [1-3]. However, premacular hemorrhage may also occur in otherwise healthy young individuals in whom no clear precipitating factor is identified, making etiological assessment more challenging. In such cases, a thorough evaluation is required to explore potential contributing factors.

Emerging evidence suggests that cannabis use may be associated with systemic cardiovascular and microvascular effects, including endothelial dysfunction, vasospasm, and blood pressure fluctuations [4-6]. However, current evidence regarding its potential impact on retinal circulation remains limited and largely observational [7,8].

Management depends on the size and location of the hemorrhage. Small or peripheral lesions may resolve spontaneously with observation. When the hemorrhage is large and centrally located, Nd:YAG laser hyaloidotomy can be used to create a small opening in the posterior hyaloid and allow drainage of blood into the vitreous cavity. Pars plana vitrectomy may be required to surgically remove the hemorrhage when it is dense, persistent, or associated with underlying retinal pathology [1-3,9].

We report a case of premacular subhyaloid hemorrhage occurring shortly after cannabis consumption in a healthy young adult, successfully treated with Nd:YAG laser hyaloidotomy.

## Case presentation

A 25-year-old man presented to the emergency department with sudden-onset decreased visual acuity in his right eye that had begun the previous day. The patient reported the sudden appearance of a dark central spot in his vision consistent with a central scotoma. His medical history was unremarkable, and he reported no known systemic diseases. He denied any history of trauma or recent Valsalva maneuvers such as coughing, vomiting, heavy lifting, or intense physical exertion. The patient reported smoking cannabis approximately two hours before the onset of visual symptoms. He described recreational cannabis use, typically smoking approximately one joint four to five times per month.

On examination, best-corrected visual acuity was 20/50 (Snellen) in the right eye and 20/20 (Snellen) in the left eye. Intraocular pressure was within normal limits bilaterally. Slit-lamp examination of the anterior segment was unremarkable.

**Figure 1:**
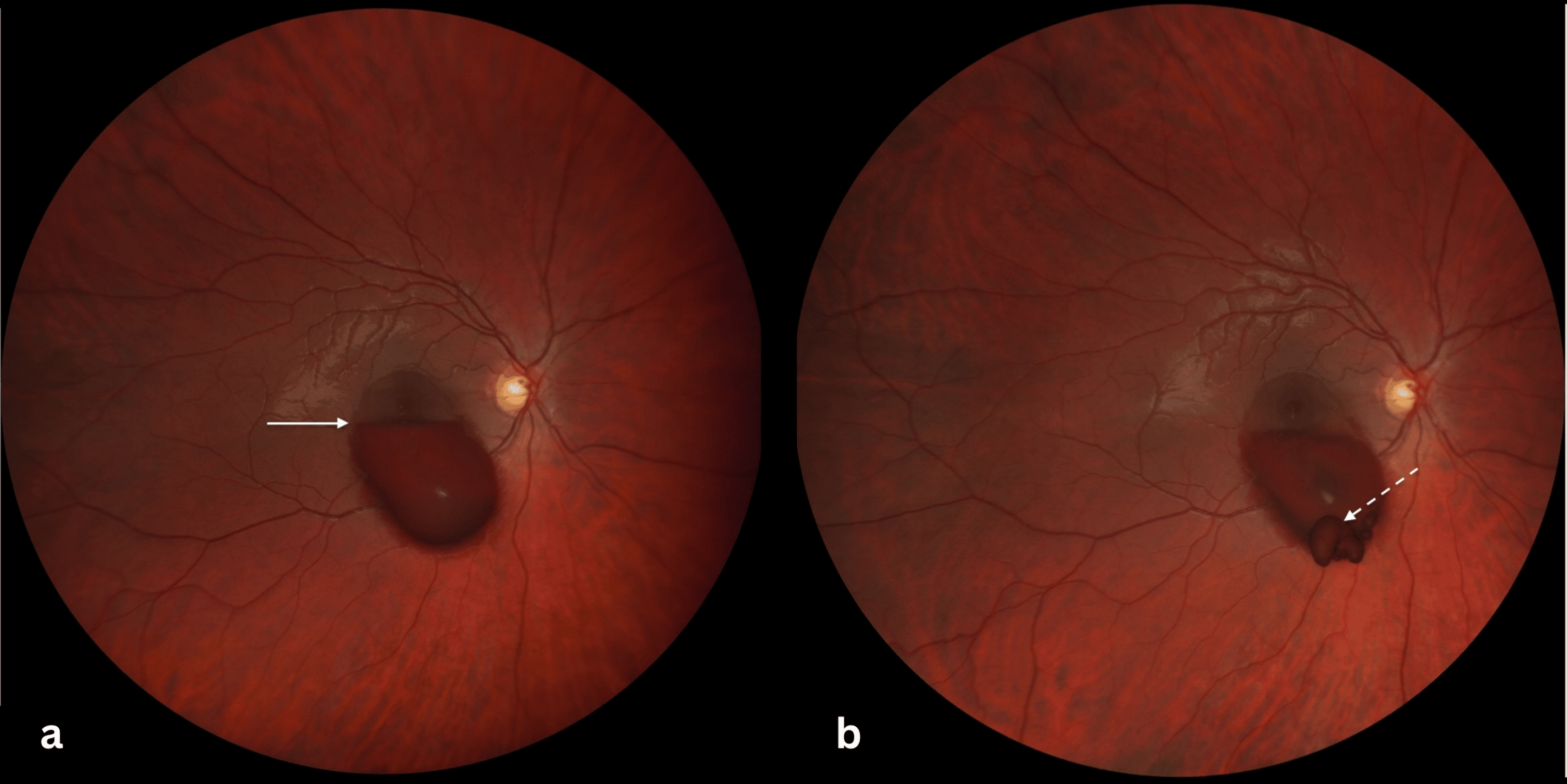
Premacular Subhyaloid Hemorrhage Before and After Nd:YAG Laser Hyaloidotomy Color fundus photographs (ZEISS Clarus 700) of the right eye showing a premacular subhyaloid hemorrhage before treatment (a) and after Nd:YAG laser hyaloidotomy (b). The hemorrhage is oval-shaped, centered on the macula, and measures approximately seven-disc diameters, with inferior extension beyond the inferior vascular arcade. A horizontal fluid–fluid level is visible (solid arrow), with a dense inferior red layer obscuring the underlying retina and a more translucent superior layer allowing partial visualization of the retinal structures. Laser energy was progressively increased, and successful posterior hyaloid perforation was achieved at 10 mJ, allowing drainage of the hemorrhage into the vitreous cavity (dashed arrow).

Fundus examination of the right eye revealed a large premacular hemorrhage measuring approximately seven-disc diameters. The hemorrhage was oval-shaped, centered on the macula, and extended inferiorly beyond the inferior vascular arcade. A horizontal fluid-fluid level was observed, with a dense red inferior layer obscuring the underlying retina, likely corresponding to erythrocyte sedimentation, and a grayish, more translucent superior layer allowing partial visualization of the retinal structures, consistent with a plasma component. No associated vitreous hemorrhage or peripheral retinal tear was observed on 360° examination (Figure 1a).

**Figure 2:**
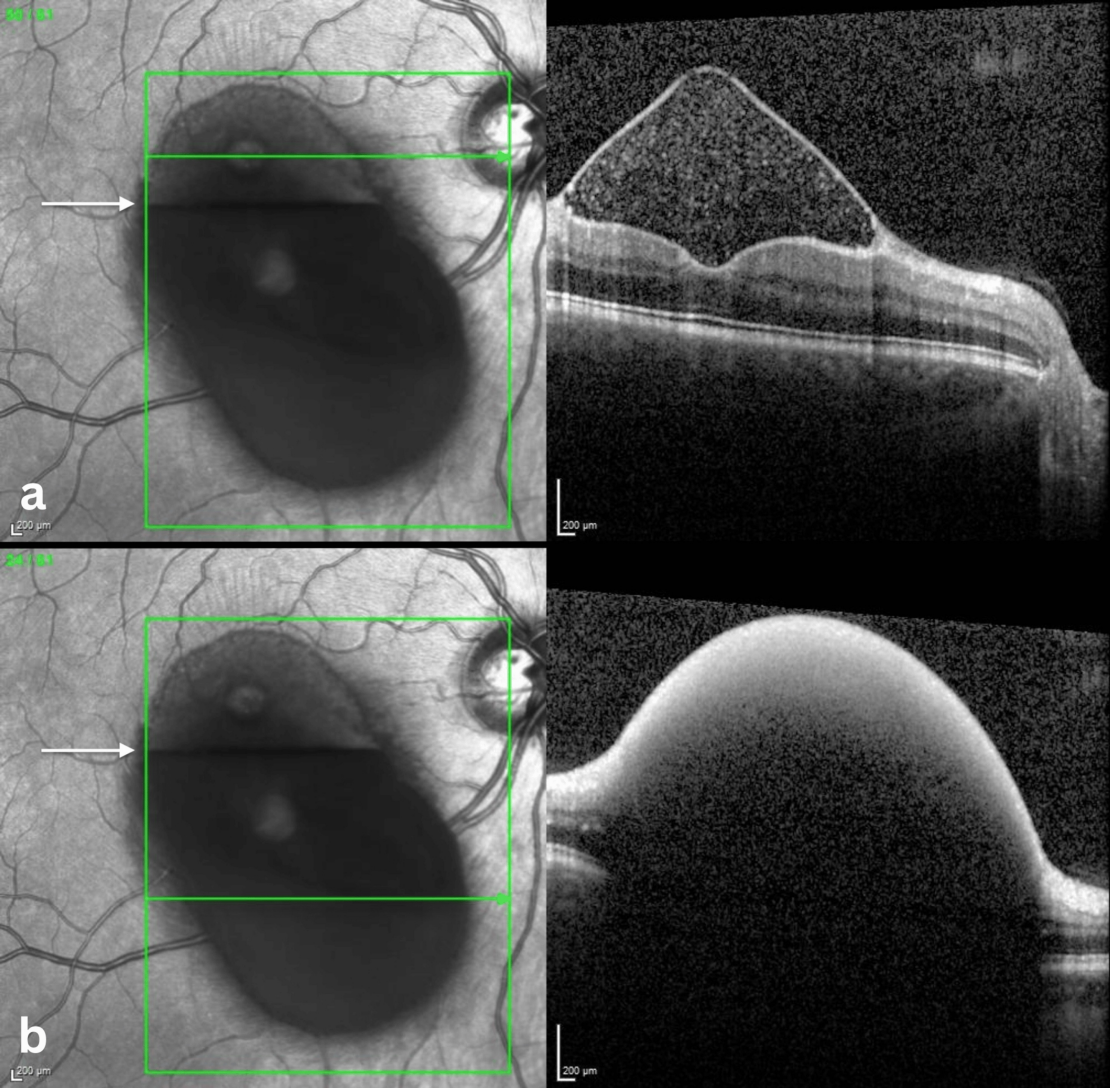
Spectral-Domain Optical Coherence Tomography of Premacular Subhyaloid Hemorrhage Spectral-domain optical coherence tomography (SD-OCT; Heidelberg) of the right eye showing two scans obtained at different levels of the premacular hemorrhage. The upper scan (a) passes above the horizontal fluid–fluid level (arrow) through the more translucent layer and demonstrates a well-demarcated triangular preretinal cavity between the internal limiting membrane (ILM) and the posterior hyaloid, containing hyperreflective material consistent with suspended blood elements. The lower scan (b) passes below the fluid–fluid level (arrow) and shows a dome-shaped, dense hyperreflective preretinal lesion producing marked posterior shadowing, which obscures visualization of the underlying retinal structures.

Spectral-domain optical coherence tomography (SD-OCT) confirmed a large preretinal collection located between the ILM and the posterior hyaloid face. The inferior portion appeared as a dome-shaped hyperreflective lesion associated with marked posterior shadowing, preventing visualization of the underlying retinal layers (Figure 2).

**Figure 3:**
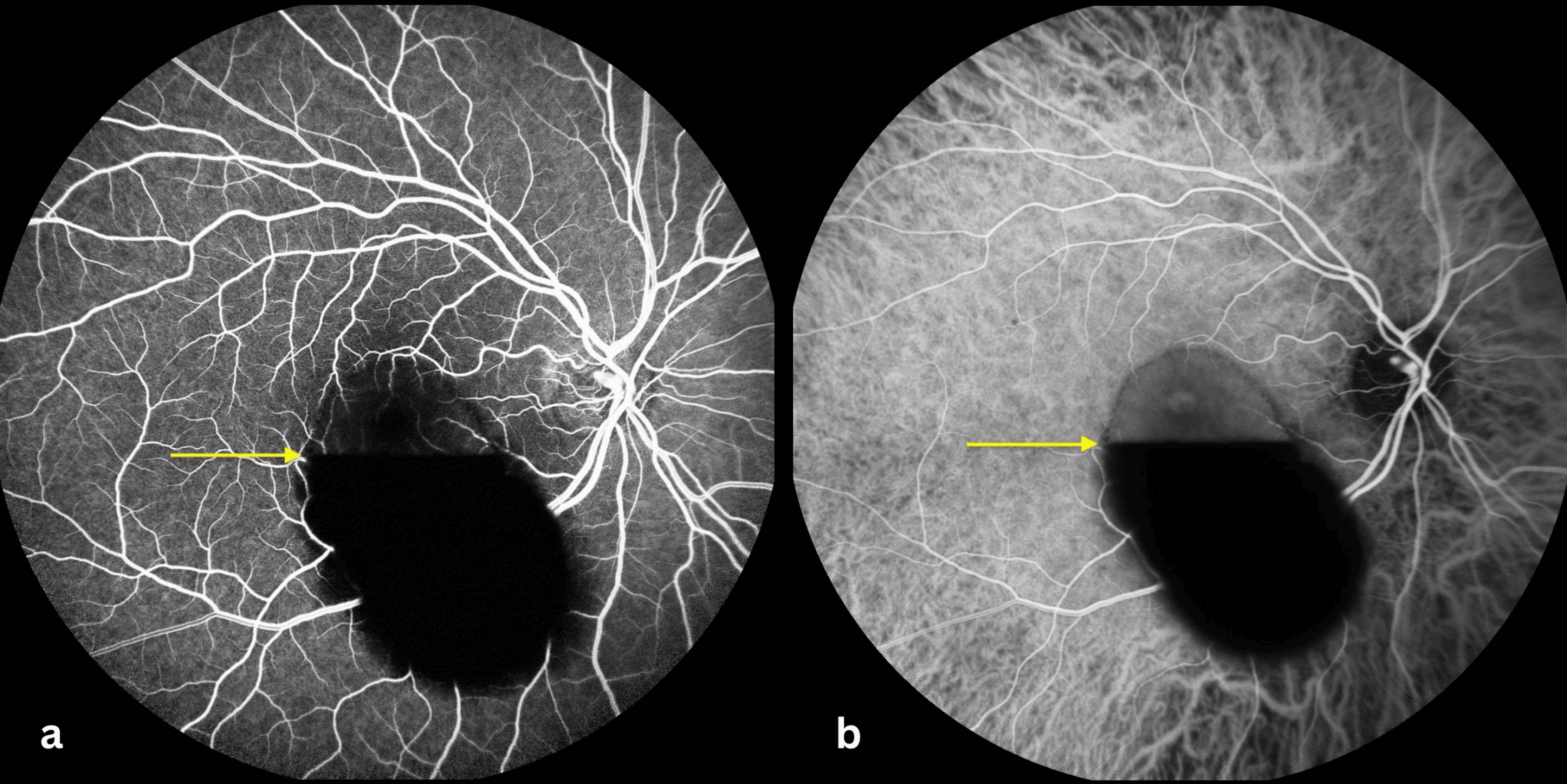
Fluorescein and Indocyanine Green Angiography of Premacular Subhyaloid Hemorrhage Fluorescein angiography (a) and indocyanine green angiography (b) of the right eye demonstrating a premacular hemorrhagic collection with a horizontal fluid–fluid level (arrow). The superior portion appears relatively translucent, allowing visualization of the underlying retinal vasculature on fluorescein angiography and the choroidal vasculature on indocyanine green angiography. In contrast, the inferior dependent portion is markedly hypofluorescent due to a masking effect from the dense hemorrhagic component, which obscures the underlying vascular structures. No evidence of retinal neovascularization, macroaneurysm, vasculitis, or other retinal vascular abnormalities is observed.

Fluorescein angiography and indocyanine green angiography demonstrated hypofluorescence corresponding to the hemorrhagic collection due to the masking effect, without evidence of retinal neovascularization, macroaneurysm, vasculitis, or other vascular abnormalities (Figure 3). Blood pressure was 128/77 mmHg. Laboratory testing excluded coagulopathy or hematologic disease.

Given the size of the hemorrhage and its central location, Nd:YAG laser hyaloidotomy was performed to facilitate rapid drainage. Two initial laser shots of 2 mJ were applied to the most elevated inferior margin of the hemorrhagic collection, away from the foveal center, and focused on the posterior hyaloid membrane. As these impacts were insufficient to achieve perforation, additional shots were delivered with progressively increasing energy levels of 4 mJ, 6 mJ, and 8 mJ. Successful perforation of the posterior hyaloid was ultimately achieved at 10 mJ, resulting in immediate drainage of blood into the vitreous cavity (Figure 1b).

The procedure was well tolerated without immediate or delayed complications. Progressive clearance of the dispersed vitreous hemorrhage was observed during follow-up, with complete resolution one week after the laser treatment. At the one-week follow-up visit, best-corrected visual acuity had improved to 20/20 (Snellen), and the patient was scheduled for a follow-up visit six months later.

## Discussion

Premacular subhyaloid hemorrhage results in sudden visual impairment due to obstruction of the fovea by accumulated blood. Spontaneous resolution is possible but may take weeks to months, with a risk of retinal toxicity related to prolonged contact between the macula and hemoglobin degradation products, as well as secondary epiretinal membrane formation [1-3]. For this reason, active intervention may be considered when the hemorrhage is large and centrally located. In our case, the premacular hemorrhage measured approximately seven-disc diameters and involved the foveal region, which supported the decision to pursue an interventional approach rather than conservative observation.

Among interventional approaches, Nd:YAG laser hyaloidotomy represents a minimally invasive option, whereas pars plana vitrectomy constitutes a more invasive surgical alternative. Nd:YAG laser hyaloidotomy may be considered in selected cases of premacular preretinal hemorrhage causing significant visual impairment, particularly when the hemorrhage is central and larger than approximately three-disc diameters. In such cases, the hemorrhagic collection can act as a protective cushion during laser application [1-3,9].

The objective is to create a small opening in the posterior hyaloid face, allowing blood to drain into the vitreous cavity and facilitating faster visual recovery. Several studies have demonstrated favorable anatomical and functional outcomes with this technique, particularly when performed early [2,3,9]. Early treatment may improve the likelihood of successful drainage, as delayed intervention can allow the hemorrhage to organize or clot, limiting blood evacuation through the laser-created opening [1].

Laser energy is typically titrated progressively, starting with low energy levels, often around 1-2 mJ, and increasing stepwise until perforation is achieved. Reported energy ranges vary across studies and individual cases, generally requiring a few millijoules but occasionally higher levels depending on membrane thickness and adherence. Gradual titration of laser energy is essential, and energy levels are generally kept below approximately 10 mJ to minimize complications such as retinal injury or macular hole formation [1,3,9].

Potential complications of Nd:YAG laser hyaloidotomy include persistent vitreous hemorrhage, incomplete or failed drainage of the hemorrhage, metamorphopsia, macular hole formation, and retinal breaks that may lead to retinal detachment [1,3,9].

Pars plana vitrectomy may be required in cases of laser failure or contraindication, very dense or organized hemorrhage, recurrence, or when associated retinal pathology necessitates surgical treatment [1,3,9].

The therapeutic decision should consider the duration of symptoms, the degree of visual impairment, the location, OCT configuration, underlying etiology, and the potential risks of laser treatment.

Beyond therapeutic considerations, the present case also raises questions regarding potential precipitating factors in otherwise healthy young individuals.

In the present case, extensive workup excluded common causes such as Valsalva maneuver, trauma, vascular abnormalities, high blood pressure or coagulopathy. The temporal relationship between cannabis consumption and symptom onset raises the possibility of a contributory vascular mechanism.

Cannabis exposure has been associated with cardiovascular and arterial complications, including endothelial dysfunction, vasospasm, platelet activation, and blood pressure fluctuations [4-6]. Associations with ischemic stroke in young adults have also been reported [6].

More specifically, ophthalmologic research has begun to examine the retinal effects of cannabinoids. A recent systematic review highlighted potential alterations in retinal physiology and vascular responses following cannabis exposure [7]. Additionally, epidemiological data have demonstrated associations between cannabis use and variations in retinal vessel diameter in young adults, suggesting possible microvascular involvement [8].

However, this observation should be interpreted with caution. Although the temporal proximity between cannabis use and symptom onset raises the hypothesis of a potential association, a causal relationship cannot be established from a single case report. At present, it also remains uncertain whether recurrence could occur during longer follow-up. A control examination at six months is planned to evaluate the long-term evolution of this case. The patient was advised to avoid further cannabis use; therefore, it will also remain unknown whether re-exposure could trigger recurrence. Even if cannabis exposure were to influence retinal vascular physiology, the dose required to induce such an effect remains unknown.

This observation contributes to the growing body of literature describing vascular events temporally associated with cannabis use. Further studies are required to clarify whether cannabis exposure constitutes an independent risk factor for retinal hemorrhagic events or represents a coincidental finding.

## Conclusions

Premacular subhyaloid hemorrhage may occur in otherwise healthy young individuals without identifiable precipitating factors. In cases of large and central premacular hemorrhage causing significant visual impairment, Nd:YAG laser hyaloidotomy can provide rapid and effective visual recovery. In younger patients, higher laser energy may occasionally be required due to increased posterior hyaloid thickness and adherence.

This case describes premacular subhyaloid hemorrhage occurring in temporal proximity to cannabis consumption in a young adult without identifiable systemic or ocular risk factors. While causality cannot be established from a single observation, the finding raises the hypothesis that cannabis-related vascular effects could potentially contribute to retinal hemorrhagic events and warrants further investigation. Careful clinical history, including recreational drug use, may therefore be valuable when evaluating otherwise unexplained retinal hemorrhage in young adults. Further observational and epidemiological studies are required to determine whether this association represents a true risk factor or a coincidental finding.
